# Effect Study of Continuous Monoculture on the Quality of *Salvia miltiorrhiza* Bge Roots

**DOI:** 10.1155/2020/4284385

**Published:** 2020-05-27

**Authors:** Hongyan Liu, Min Niu, Shu Zhu, Fang Zhang, Qian Liu, Yang Liu, Ruohan Liu, Yongqing Zhang

**Affiliations:** ^1^Experimental Center, Shandong University of Traditional Chinese Medicine, Jinan 250355, China; ^2^Jiangsu College of Nursing, Huai'an 223005, China; ^3^Basic Medicine, Shandong University of Traditional Chinese Medicine, Jinan 250355, China; ^4^School of Pharmacy, Shandong University of Traditional Chinese Medicine, Jinan 250355, China

## Abstract

High-efficiency monoculture severely inhibits the growth of *Salvia miltiorrhiza* Bge and decreases the yield and quality of crude drug, thus resulting in serious economic losses in China. Here, we selected four replanted field soils with 1, 2, 3, and 4 years of monoculture history to investigate the influence of continuous monocropping soil on the property of medicinal materials by pot experiments. Results showed that the commodity appearance and active ingredient contents of *Salvia miltiorrhiza* were significantly affected by soil with different continuous monocropping years. Along the time series of plantation soil, the diameter of main roots, weight of fresh roots, and total contents of hydrophilic and lipophilic components demonstrated a decline tendency. With the method of PCA, the property of medicinal materials affected by continuous monocropping soil was evaluated by the following formula: *F* = (0.3762 × F1 + 0.2320 × F2 + 0.1913 × F3 + 0.0994 × F4)/0.8989. Eventually, crude drug properties ranked according to comprehensive scores were as follows: CK (0.380) > 1 year (0.360) > 2 years (0.348) > 3 years (0.337) > 4 years (0.245). For the medicinal plant of *Salvia miltiorrhiza* Bge, continuous monocropping soil had significant effects on the property of *Salvia miltiorrhiza* and should be ameliorated by some measures. The results provide support for the optimal continuous cropping year selection and continuous cropping obstacle abatement of *Salvia miltiorrhiza* Bge.

## 1. Introduction

The roots and rhizome of *Salvia miltiorrhiza* Bge, recorded in the Chinese Pharmacopoeia with the name of Danshen, are commonly used to treat a wide variety of ailments including cardiovascular and cerebrovascular diseases, liver diseases, and diabetes mellitus in traditional Chinese medicine [1]. At present, due to its multiple pharmacological effects such as antibacterial, anti-inflammatory, and antiallergic activity [2], it has been developed into more than thirty pharmaceutical dosage forms and its consumption in the world has exceeded 20 million kilograms per year [3]. With such a particularly strong and rapid upsurge in clinical demand, traditional plantations of this medical plant can no longer meet the need of demand growth. In order to meet the surging market demand, continuous cropping of *Salvia miltiorrhiza* Bge on the same area with or without short rotation increased dramatically. However, high-efficiency monoculture often leads to continuous cropping obstacle, which eventually causes poor growth, low yield, and serious insect disease. In China, this problem has resulted in severe economic losses and hindered the sustainable development of *Salvia miltiorrhiza* industry.

Consecutive monoculture problem, popularly known as replant disease or soil sickness, usually leads to a series of problems such as inhibition of plant growth, reduction in the yield and quality, and exhaustion of soil fertility [4]. As to the nature and mechanism of this phenomenon, soil deterioration induced by physiochemical property disorders, soil microbe, and accumulation of autotoxic chemicals were proven to be the major causes in the continuous cropping obstacles of some crops [5]. A growing body of studies has confirmed the fact that with the increase in planting years, the properties of continuous cropping soil became more and more deteriorated and then exerted some effect on the crop yield and quality. For example, a document recorded that replanted coffee fields with 4, 18, 26, and 57 years resulted in the significant alteration of soil microbial community memberships and structures, which might lead to the poor growth of coffee plants in pots and decline of coffee yields in fields [6]. Similar results also happened on *Pseudostellaria heterophylla* [7], potato [8], and some other annual or perennial plants.

Our previous studies have also confirmed that some phenolic acids released from two-year consecutive planting of *Salvia miltiorrhiza* Bge roots to the soil contribute to the inhibition of its self-growth [9]. Hence, we hypothesize that continuous cropping soil has a direct or indirect effect on the growth of *Salvia miltiorrhiza* Bge and then results in the change of the biomass, yield, and quality of crude drug. Therefore, in this study, a pot experiment with soil from the field that had undergone continuous cropping for *Salvia miltiorrhiza* Bge was designed to evaluate the influence of continuous cropping on the quality of *Salvia miltiorrhiza*. In traditional cognition, the quality of *Salvia miltiorrhiza* mainly consisted of commodity appearance and active ingredient contents or biomass, so these kinds of indices were determined in this study.

## 2. Materials and Methods

### 2.1. Soil Sources

Two types of soil were used in this experiment. The first type was, respectively, collected from where *Salvia miltiorrhiza* Bge had been cultivated for a period of 4 years, 3 years, 2 years, and 1 year in the Germplasms Nursery of Shandong University of TCM (SDCM), in March 2016. The second type is the control and collected from where *Salvia miltiorrhiza* Bge had never been planted in the same field. The soil was then further sieved through a 2 mm mesh to remove debris and root tissues. Samples of rhizosphere soils of *Salvia miltiorrhiza* Bge were collected by pulling plants from the soil and shaking soil off from plant roots [2] for allelopathic plant cultivation. The collected sandy soil was soaked in two same amounts of water, and then, the supernatants were tested with a pH meter. The blank soil sample was tested weak alkaline with the pH value of 8.01. For the sandy sample soil, with the continuous cropping years increased, the acidity of sandy soil samples enhanced with the pH value of 7.81, 7.64, 7.33, and 7.02, respectively.

### 2.2. Plant Growth

The collected soil was immediately put into polyethylene pots (*Ø* = 12.5 cm; 25pots, each containing 750 g soil). The robust and uniform semiannual *Salvia miltiorrhiza* seedlings were selected and planted in each pot and allowed to grow for eight months under the same management conditions. All of the pots were kept in bright sunlight, allowing the medicinal plants to grow naturally with normal watering and farmyard manure just like those planted in the field. Twenty pots were maintained per treatment as replicates.

### 2.3. Commodity Appearance and Biomass of the Roots of *Salvia miltiorrhiza* Bge

During the pot experiment, plants were carefully uprooted in the root harvest period of late October, keeping the root system intact, and the roots were collected. Firstly, the biomass of fresh *S. miltiorrhiza* roots was recorded; then, the collected roots were air-dried, finely powdered for chemical content determination. Voucher specimens were deposited in the herbarium of Shandong University of TCM (SDCM).

### 2.4. Determination of Chemicals in *S. miltiorrhiza* Roots

#### 2.4.1. Standards and Reagents

Eight reference standards, including rosemary acid (20120809), salvianolic acid B (PA0418RA13), lithospermic acid (PF225SA14), dihydrotanshinone I (KN1113GU14), tanshinone II (20130111), danshensu sodium (YY90062), tanshinone I (110867-200406), and cryptotanshinone (110852-200806) with purity not less than 98%, were obtained from Yuanye Biological Technology Company (Beijing, China). All chemical reagents were analytical grade and purchased from Tianjin Fu-Yu Chemical Reagent Factory.

#### 2.4.2. Preparation of Standard Reference Solutions

Accurate weight of the reference compound was dissolved in 70% methanol or methanol, respectively, to yield eight individual standard stock solutions.

The hydrophilic working standard references including danshensu sodium, salvianolic acid B, rosemary acid, and lithospermic acid were prepared by diluting mixed standard stock solutions with 70% methanol to get six different concentrations for calibration curves. The lipophilic standard references including dihydrotanshinone I, tanshinone IIA, cryptotanshinone, and tanshinone I were also prepared by diluting mixed standard stock solutions with methanol to obtain six different concentrations for calibration curves.

All the standard stock and working solutions were prepared in dark-brown calibrated flasks and stored at 4°C.

#### 2.4.3. Preparation of Sample Solutions

The air-dried material was powdered to a homogeneous size using an electrical blender and sieved through a 40-mesh sieve. For quantitative analysis, the accurately weighed sample (0.25 g) was suspended in 25 mL of 75% methanol in a conical flask with a cover. The weight of the flask along with the contents was recorded, and the mixture was ultrasonically extracted at 50°C for 50 min. After ultrasonic treatment, the original solvent weight was restored by adding 75% methanol. Subsequently, the extract was filtered through a 0.45 *μ*m membrane for component analysis.

The hydrophilic component solution was prepared following the above method with methanol as the solvent.

#### 2.4.4. Apparatus and Chromatographic Conditions

Chromatography was performed on Agilent 1260 Series HPLC equipment (Agilent Corporation, USA) with a ZORBAXSB-C_18_ column (4.6 mm × 250 mm, 5 *μ*m) at 30°C. The mobile phase consisting of acetonitrile (A) and 0.1% aqueous phosphoric acid (B) in a gradient elution mode for hydrophilic component determination was as follows: 0~20 min, 3%-23% A; 20~35 min, 23%-25% A; 35~40 min, 25% A; 40~50 min, 25%-90% A; and 50~60 min, 90% A. The flow rate was 1.0 mL·min^−1^, the sample injection volume was 10 *μ*L, and the detection wavelength was set at 286 nm.

The mobile phase in a gradient elution mode for lipophilic component determination was as follows: 0~20 min, 20%-60% A; 20~50 min, 60%-80% A; and 50~55 min, 80% A. The flow rate was 1.0 mL·min^−1^, the sample injection volume was 10 *μ*L, and the detection wavelength was set at 270 nm.

#### 2.4.5. Validation of the HPLC Method

The calibration curve for each compound was established by plotting the peak area (*Y*) versus concentration (*x*) of each analyte. Under optimal chromatographic conditions, the diluted six appropriate concentrations of working standard solutions were injected in triplicate, and their regression equations were calculated in the form of *Y* = *Ax* + *B*.

The intraday and interday variability measurement was utilized to assess the precision of the analytical method. The intraday variation was determined by analyzing the six replicates of standard solutions on the same day while the interday variation was determined on three consecutive days. Stability was tested with an extract solution of one sample at 0, 2, 4, 8, 12, 24, 48, and 72 h within 3 days. All solutions were kept at 4°C before analysis.

#### 2.4.6. Continuous Cropping Effect Evaluation with Principal Component Analysis (PCA)

In the study of many indexes, the phenomenon of information overlap of observational data exists because of too many variables and possible correlations between them. So we used principal component analysis to search for several factors which have no correlation to represent all the variables. In this study, the values of the biomass including the diameter of main roots, length of main roots, and fresh weight and eight analyte contents of the roots of *Salvia miltiorrhiza* Bge were selected for principal component analysis by SPSS 19.0 (SPSS Inc., Illinois), and the comprehensive scores were calculated to evaluate the continuous cropping effects.

#### 2.4.7. Statistical Analysis

One-way analysis of variance (ANOVA) procedures were performed in the statistical software SPSS 19.0 (SPSS Inc., Illinois) to assess the effects of different cropping years of plantation soil on biomass and chemical contents of the roots of *Salvia miltiorrhiza* Bge. All the data were represented as means ± standard deviation, and means were compared using Duncan's multiple range test at a 5% level.

## 3. Results

### 3.1. Effects of Continuously Cultivated Soils on Biomass of *Salvia miltiorrhiza*

Table 1 clearly demonstrated that continuously cultivated soil had a significant impact on the biomass of *S. miltiorrhiza* roots. Along the time series of plantation soil and compared to the control, the diameter of main roots decreased by 12.63%, 18.99%, 32.95%, and 36.33%, the length of main roots decreased by 31.70%, 39.60%, 33.76%, and 37.25%, and the fresh weight or the yield decreased by 24.9%, 38.6%, 48.9%, and 53.8%, respectively. The facts indicate that continuous planting would cause yield and quality decline of *Salvia miltiorrhiza*. In addition, the treated plantation soil showed no significant effect on the length of main roots.

### 3.2. Method Validation

**Figure 1:**
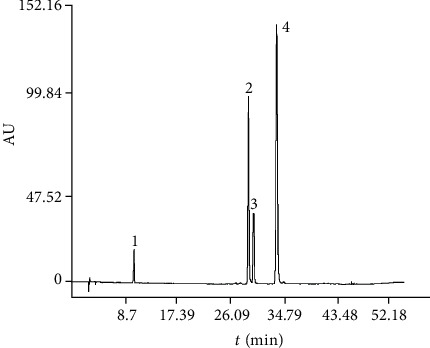
The HPLC chromatogram of the hydrophilic reference compounds of *Salvia miltiorrhiza*. The peaks are 1 (danshensu sodium), 2 (rosemary acid), 3 (lithospermic acid), and 4 (salvianolic acid B).

**Figure 2:**
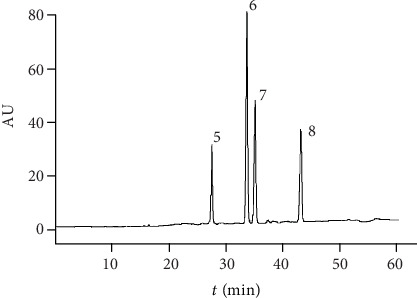
The HPLC chromatogram of the lipophilic reference compounds of *Salvia miltiorrhiza*. The peaks are 5 (dihydrotanshinone I), 6 (cryptotanshinone), 7 (tanshinone I), and 8 (tanshinone IIA).

The regression equations of the eight analytes are shown in Table 2. All the marker reference showed good linearity (*R*^2^ > 0.9991) in a relatively wide concentration range. The overall stability and repeatability variations were less than 3%. The HPLC chromatogram of the eight reference compounds is shown in Figures 1 and 2.

### 3.3. Effects of Continuously Cultivated Soils on the Compositions of *Salvia miltiorrhiza*

**Figure 3:**
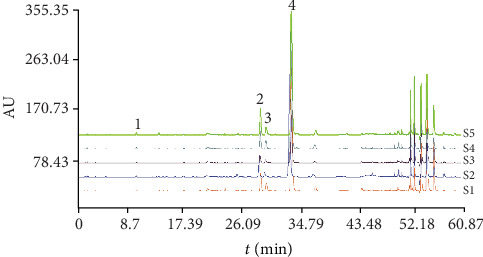
The HPLC chromatogram of hydrophilic components in samples from different treatment groups. The peaks are 1 (danshensu sodium), 2 (rosemary acid), 3 (lithospermic acid), and 4 (salvianolic acid B).

**Figure 4:**
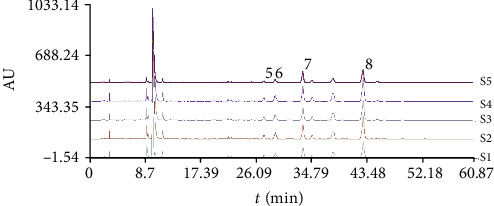
The HPLC chromatogram of lipophilic components in samples from different treatment groups. The peaks are 5 (dihydrotanshinone I), 6 (cryptotanshinone), 7 (tanshinone I), and 8 (tanshinone IIA).

Figures 3 and 4 showed the HPLC chromatogram of hydrophilic and lipophilic components in samples from different treatment groups, respectively. As shown in Tables 3 and 4, different groups of continuously cultivated soil had a significant impact on the hydrophilic and lipophilic component contents of *Salvia miltiorrhiza* compared with the control. With the exception of danshensu sodium, the contents of rosemary acid, lithospermic acid, and salvianolic acid B in *S. miltiorrhiza* roots showed significant difference among treated groups, while the contents of dihydrotanshinone I, cryptotanshinone, tanshinone I, and tanshinone IIA varied significantly among different treated groups (*P* < 0.05). For hydrophilic components or lipophilic ingredients, the total contents of the 8 determined ingredients reduced with the increase in continuous cropping years of soil.

### 3.4. Property Assessment Results of Crude Drug

The data including biomass and component contents from all of the treatments were analyzed using PCA to identify the primary factors. The PCA results demonstrated that the four principal components (F1, F2, F3, and F4) contained 89.88% information of the original indexes from the results of the eigenvalue and variance contribution rate of the principal component (Table 5) and factor load matrix (Table 6).

The first principal component F1 (37.62%) reflected the comprehensive variation information of danshensu sodium, rosemary acid, lithospermic acid, salvianolic acid B, and dihydrotanshinone I. The second principal component F2 (23.20%) reflected the comprehensive variation information of cryptotanshinone, tanshinone I, tanshinone IIA, diameter of main roots, and fresh root weight. The third principal component F3 (19.13%) revealed comprehensive variation information of dihydrotanshinone I, tanshinone IIA, and length of main roots. The forth component F4 (9.94%) reflected that the diameter of main roots contributed the maximum value.

The column elements of F1, F2, F3, and F4 from the factor load matrix (Table 6) are divided by the corresponding eigenvalue from Table 5, and the results are then taken using the arithmetic square root. The ultimate results were principal component feature vectors, which were also the coefficient of the linear equation between the four principal components and each variable. The linear equations are as follows:

(1) $$\begin{array}{c} \text{F}1=0.148X_{1}-0.136X_{2}+0.151X_{3}+0.136X_{4}-0.090X_{5}-0.041X_{6}-0.056X_{7}+0.061X_{8}+0.065X_{9}+0.052X_{10}+0.070X_{11},\\ \text{F}2=-0.008X_{1}+0.015X_{2}+0.026X_{3}+0.089X_{4}+0.041X_{5}+0.135X_{6}+0.186X_{7}+0.107X_{8}-0.064X_{9}-0.107X_{10}+0.141X_{11},\\ \text{F}3=0.051X_{1}-0.091X_{2}+0.159X_{3}+0.032X_{4}+0.165X5+0.107X6+0.033X7-0.156X8-0.104X9+0.134X_{10}-0.040X_{11},\\ \text{F}4=-0.067X_{1}-0.100X_{2}-0.040X_{3}+0.020X_{4}+0.012X_{5}+0.147X_{6}+0.049X_{7}+0.042X_{8}+0.229X_{9}+0.016X_{10}-0.123X_{11}. \end{array}$$

The scores of the tested groups on the four principal components, which were the weight for the linear combination equation, were calculated by using the input standardized raw data into the above linear equations, and the comprehensive model was established by the following formula:

(2) $$\begin{array}{c} F=\frac{0.3762\times \text{F}1+0.2320\times \text{F}2+0.1913\times \text{F}3+0.0994\times \text{F}4}{0.8989}. \end{array}$$

Finally, with the aid of the eigenvector of eleven variance, the comprehensive scores of property assessments of crude drug for the control, 1-year continuous monocropping soil, 2-year continuous monocropping soil, 3-year continuous monocropping soil, and 4-year continuous monocropping soil groups were 0.380, 0.360, 0.348, 0.337, and 0.245, respectively. So the magnitude sequence of the comprehensive score for *S. miltiorrhiza* roots were the control > 1 − year continuous cropping soil planted > 2 − year continuous cropping soil planted > 3 − year continuous cropping soil planted > 4 − year continuous cropping soil planted.

## 4. Discussion

In commercial trade, the profit of *Salvia miltiorrhiza* was closely associated with its commodity appearance, active ingredient contents, and yield. So the high yield and quality are the primary goal of medicinal plant growers. In addition, the fresh weight index of *Salvia miltiorrhiza* can represent its yield, and the hydrophilic and lipophilic component contents which were stipulated in the Chinese Pharmacopoeia can represent its quality to some extent, while the main root length and diameter indicators could reflect commodity appearance to a certain extent. Till now, the active ingredients of *Salvia miltiorrhiza* mainly consisted of two categories of lipophilic and hydrophilic compounds. Among the former, tanshinone and its derivatives display a range of biological activities including antibacterial and antioxidant effects and prevention of angina pectoris and myocardial infarction [10, 11], While in the latter group, the representative lipophilic diterpenoidal quinone compounds showed obvious antioxidant capacity [10, 11]. Thus, the contents of active ingredients are closely related to clinical efficacy and are one of the decisive factors affecting the commodity price of *Salvia miltiorrhiza*. Thus, those indicators of the main root length, diameter, component content, and fresh weight could comprehensively reflect the overall quality of *Salvia miltiorrhiza* together.

However, in China, *Salvia miltiorrhiza* Bge was one of about 70% of medicinal plant species with tuber roots suffering from consecutive monoculture problems and its quality was not always very palatable [7]. Our different-year soil experiment revealed typical growth inhibition effects on the diameter, length, and fresh weight of *Salvia miltiorrhiza* Bge roots and no influence on the length of main roots. When it comes to the content change of the eight lipophilic and hydrophilic compounds, there was no regularity with increasing years of monoculture. Because there was no regularity to explore for those indicators and a single indicator could not reflect the comprehensive effects induced by continuous cropping soil on *Salvia miltiorrhiza* Bge, a strategy must be adopted to evaluate those regularly changed or irregularly changed indicators from the whole. So a linear feature extraction was designed by using the principal component analysis method to calculate the comprehensive score of all those indicators in each test group. The PCA statistical method, with the merits of simplifying the complexity in high-dimensional data while retaining trends and patterns, was an appropriate strategy to evaluate those indicators including the diameter, length, and fresh weight of roots and contents of danshensu sodium, rosemary acid, lithospermic acid, salvianolic acid B, dihydrotanshinone I, cryptotanshinone, tanshinone I, and tanshinone IIA comprehensively. The results of the final comprehensive scores showed that the overall quality of *Salvia miltiorrhiza* declined along the time series of 1, 2, 3, and 4 years of continuous planting.

Besides structural imbalance of microorganisms, autotoxin accumulation mainly induced by plant residues and root exudates was also popularly accepted as an important factor contributing to continuous cropping obstacle [12]. In actual production, after the harvest of *Salvia miltiorrhiza* Bge, the aerial parts and taproots were removed, while some fibrous root residuals were left behind in the planting soil, which will be decomposed, releasing allelochemicals and changing soil microecological balance. These chemicals may contain some water-soluble substances, which are regarded to contribute to replant failure at the same time. With the increase in the period of continuous cultivation, more and more allelochemicals accumulate in the soil and soil microecological imbalance becomes more and more serious. Then, autotoxic substance mediation, along with soil microecological balance, would disturb the successive season planting. So the yield and quality of *Salvia miltiorrhiza* in our study demonstrated downward trends with the period of continuous cultivation of soil increased. The same findings also concurred with a previous report of *Angelica sinensis* (Oliv.) Diels. Pot experiments showed that the root yield, content of essential oils, and ethanol extract of *Angelica sinensis* (Oliv.) Diels planted in continuous cropping soil declined significantly compared with the control [5]. Another study also demonstrated that continuous *Angelica sinensis* (Oliv.) Diels cropping significantly decreased the plant height, main root length, and biomass of *Angelica Sinensis* (Oliv.) Diels plants during the vegetative period and then led to lower yields [13].

Recently, with the advance of modern agriculture, serious continuous cropping obstacles occurred in more and more medicinal plant cultivation. Seeking effective measures to confront this problem has attracted the attention of many scholars. Till now, crop rotation, tillage, and fertilization are proven to be the effective strategies to overcome continuous cultivation obstacle in agricultural production through altering soil properties [14]. These kinds of feasible measures will be applied to *Salvia miltiorrhiza* cultivation in our further work.

## 5. Conclusions

The continuous cropping obstacles can decrease the yield and quality of many medicinal plants. Although the reason was complex, the soil microbes and autotoxic substances released into the soil were popularly proven to be the major determinants. With the continuous cropping years increased, the soil physiochemical property changed differently and then would have a different effect on the quality and yield of medicinal materials. Pot experiments proved that 1-, 2-, 3-, and 4-year continuous cropping soil not only reduced the quality and yield of *Salvia miltiorrhiza* Bge but also affected its active ingredient content obviously. Because a single indicator could not evaluate the influence wholly, the PCA method was applied to establish linear equations to analyze those multiple indicators. The results provide support for the optimal continuous cropping year selection and continuous cropping obstacle abatement of *Salvia miltiorrhiza* Bge.

## Figures and Tables

**Table 1:** Effects of continuous cropping soil on the biomass of *S. miltiorrhiza* roots (*n* = 50).

Groups	Diameter of main roots (cm)	Length of main roots (cm)	Fresh weight of roots (g)
Control	12.11 ± 1.43A	15.33 ± 0.81A	62.07 ± 2.74A
1-year continuous cropping soil	10.58 ± 1.01B	10.47 ± 0.64B	46.60 ± 2.03B
2-year continuous cropping soil	9.81 ± 0.59B	9.26 ± 1.23B	38.14 ± 1.45C
3-year continuous cropping soil	8.12 ± 1.32C	10.15 ± 1.38B	31.75 ± 1.12D
4-year continuous cropping soil	7.71 ± 0.91C	9.62 ± 0.98B	28.71 ± 1.31E

Note: the values denoted by different letters within the same column represent significant difference at the 0.05 level as compared with various concentration treatment groups.

**Table 2:** Regression data of eight analytes.

Analyte	Regression equation	*R* ^2^	Linear range (*μ*g/mL)
Lithospermic acid	*Y* = 48.20*x* − 29.45	0.9916	1.43~28.64
Salvianolic acid B	*Y* = 216.21*x* + 33.54	1	17.43~348.50
Rosemary acid	*Y* = 116.24*x* − 14.02	0.9991	1.81~36.20
Danshensu sodium	*Y* = 14.08*x* + 8.89	0.9993	0.30~5.90
Dihydrotanshinone I	*Y* = 225.76*x* + 12.247	1	1.54~30.86
Tanshinone IIA	*Y* = 69.21*x* + 7.62	0.9999	0.52~10.34
Cryptotanshinone	*Y* = 169.29*x* + 16.74	1	1.32~26.48
Tanshinone I	*Y* = 8.65*x* + 2.91	0.9998	0.066~13.18

**Table 3:** Effects of continuous cropping soil on *Salvia miltiorrhiza* hydrophilic compositions ($\bar{x}\pm s$, *n* = 3).

Groups	Contents (%)				
	Danshensu sodium	Rosemary acid	Lithospermic acid	Salvianolic acid B	Total content
Control	0.035 ± 0.0013A	0.434 ± 0.0056A	0.328 ± 0.0016A	4.421 ± 0.0167A	5.218
1-year continuously cultivated soil	0.051 ± 0.0006B	0.416 ± 0.0017B	0.298 ± 0.0087B	4.217 ± 0.0088B	4.982
2-year continuously cultivated soil	0.052 ± 0.0025B	0.229 ± 0.0025C	0.262 ± 0.0027B	4.433 ± 0.0011C	4.976
3-year continuously cultivated soil	0.044 ± 0.0005B	0.437 ± 0.0009A	0.246 ± 0.0008C	2.044 ± 0.0039B	2.771
4-year continuously cultivated soil	0.053 ± 0.0016B	0.588 ± 0.0007D	0.228 ± 0.0007D	1.941 ± 0.0017D	2.809

Note: the values denoted by different letters within the same column represent significant difference at the 0.05 level as compared with various concentration treatment groups.

**Table 4:** Effects of continuously cultivated soils on *Salvia miltiorrhiza* lipophilic constituents ($\bar{x}\pm s$, *n* = 3).

Groups	Contents (%)				
	Dihydrotanshinone I	Cryptotanshinone	Tanshinone I	Tanshinone IIA	Total content
Control	0.217 ± 0.0065A	0.564 ± 0.0060A	0.268 ± 0.0015A	0.305 ± 0.0013A	1.354
1-year continuously cultivated soil	0.262 ± 0.0005B	0.481 ± 0.0001B	0.242 ± 0.0027B	0.258 ± 0.0002B	1.243
2-year continuously cultivated soil	0.302 ± 0.0004C	0.331 ± 0.0019C	0.158 ± 0.0004C	0.128 ± 0.0009C	0.92
3-year continuously cultivated soil	0.231 ± 0.0012AB	0.252 ± 0.0006D	0.099 ± 0.0016D	0.082 ± 0.0002D	0.664
4-year continuously cultivated soil	0.171 ± 0.0011D	0.165 ± 0.0012E	0.076 ± 0.0005E	0.052 ± 0.0011E	0.464

Note: the values denoted by different letters within the same column represent significant difference at the 0.05 level as compared with various concentration treatment groups.

**Table 5:** Eigenvalue and variance contribution rate of principal components.

Principal component	Eigenvalue	Variance contribution rate (%)	Cumulative variance contribution rate (%)
F1	4.138	37.617	37.617
F2	2.552	23.202	60.819
F3	2.105	19.132	79.951
F4	1.093	9.935	89.886

**Table 6:** The factor load matrix of variables.

Variable	F1	F2	F3	F4
*X*_1_				
Danshensu sodium	0.906	-0.038	0.225	-0.212
*X*_2_				
Rosemary acid	-0.834	0.07	-0.396	-0.314
*X*_3_				
Lithospermic acid	0.926	0.127	0.26	-0.126
*X*_4_				
Salvianolic acid B	0.832	0.427	0.14	0.063
*X*_5_				
Dihydrotanshinone I	-0.553	0.196	0.722	0.038
*X*_6_				
Cryptotanshinone	-0.254	0.652	0.469	0.462
*X*_7_				
Tanshinone I	-0.341	0.894	0.142	0.156
*X*_8_				
Tanshinone IIA	0.373	0.514	-0.682	0.132
*X*_9_				
Diameter of main roots	0.399	-0.308	-0.455	0.72
*X*_10_				
Length of main roots	0.318	-0.513	0.587	0.051
*X*_11_				
Fresh root weight	0.431	0.681	-0.176	-0.388

## Data Availability

The raw/processed data required to reproduce these findings cannot be shared at this time as the data also forms part of an ongoing study.
